# A qualitative study of perceived social barriers to care for eating disorders: Perspectives from ethnically diverse health care consumers

**DOI:** 10.1002/eat.20755

**Published:** 2009-10-05

**Authors:** Anne E Becker, Adrienne Hadley Arrindell, Alexandra Perloe, Kristen Fay, Ruth H Striegel-Moore

**Affiliations:** 1 Department of Global Health and Social Medicine, Harvard Medical SchoolBoston, Massachusetts; 2 Eating Disorders Clinical and Research Program, Department of Psychiatry, Massachusetts General HospitalBoston, Massachusetts; 3 Eliot Pearson Department of Child Development, Tufts UniversityMedford, Massachusetts; 4 Department of Psychology, Wesleyan UniversityMiddletown, Connecticut

**Keywords:** ethnicity, stereotypes, stigma, eating disorders, access to care

## Abstract

**Objective:**

The study aim was to identify and describe health consumer perspectives on social barriers to care for eating disorders in an ethnically diverse sample.

**Method:**

We conducted an exploratory secondary analysis of qualitative data comprising transcripts from semi-structured interviews with past and prospective consumers of eating disorder treatment (*n* = 32). Transcripts were inputted into NVivo 8 for coding, sorting, and quantifying thematic content of interest within strata defined by ethnic minority and non-minority participants. We then examined the influence of key social barriers—including stigma and social stereotypes—on perceived impact on care.

**Results:**

The majority of respondents (78%) endorsed at least one social barrier to care for an eating or weight concern. Perceived stigma (or shame) and social stereotyping—identified both within social networks and among clinicians—had adversely impacted care for 59% and 19% of respondents, respectively.

**Discussion:**

Social barriers to care for eating and weight related concerns may be prevalent in the U.S. and impact both ethnic minority and non-minority health care consumers. © 2009 by Wiley Periodicals, Inc. (Int J Eat Disord 2010;)

## Introduction

Despite evidence that a large percentage of individuals with an eating disorder do not access specialty care,^1^ little is known about specific barriers to accessing care for eating disorders. Numerous reports indicate that eating disorders are frequently unrecognized in clinical settings,^2^–^6^ and other studies suggest that appropriate care is not initiated even when eating disorder symptoms do come to clinical attention.^7^,^8^

Although diverse societal, health-system-based, and individual factors generally influence mental health service utilization,^9^,^10^ utilization patterns for an eating disorder may be distinguished by additional barriers to care.^11^ For example, psychological factors relating to motivation, denial, and limited insight have been identified as having an adverse impact on help-seeking and therapeutic engagement,^12^–^15^ and may also undermine clinical detection.^11^,^16^ The ego-syntonic nature of core eating disorder symptoms, such as severe weight loss or purging (at least in the early stages of the illness), may interfere with an individual's willingness to seek treatment. Both the specific eating disorder diagnosis as well as impairment have been found to be associated with mental health service utilization among individuals with eating disorders.^11^ However, social factors—for example, cultural practices, social norms, or socially-based inequities in health resource distribution—impeding access to care for eating disorders have comparatively less visibility in the eating disorders literature. These social factors appear salient to understanding reported ethnic disparities in receipt of eating disorders care.

## Ethnic Disparities in Utilization of Eating Disorders Care

Ethnic disparities have been well documented for general health care access, treatment delivery, and outcomes in the United States.^17^,^18^ These inequities are especially germane for mental health, for which racial minorities are at risk for non-detection in primary care practices.^19^ Several studies also support that ethnicity may be a risk correlate of reduced access to care for an eating disorder in the U.S.^20^,^21^ Concerns about ethnic disparities in service access for an eating disorder have been raised for ethnic minority patients in the UK^22^–^24^ and for immigrants to Australia as well.^11^ In one community-based sample of ethnically diverse women with eating disorders, a majority did not receive care for their eating disorder; African-American women, in particular, were significantly less likely to receive care for an eating disorder when compared with white women.^25^ Likewise, two other studies have shown ethnicity-based disparities in the treatment of binge eating disorder (BED), with black women significantly less likely than white women to have received treatment.^26^,^27^

Clinician expectations about ethnicity and differential risk that demonstrably contribute to disparities in care and accurate diagnosis for other mental disorders may also be relevant to eating disorders.^28^ Clinician-based factors that differentially impact therapeutic decisions for ethnic minority patients may relate to clinician unfamiliarity with ethnic diversity in help-seeking and manifestation of an eating disorder.^29^–^31^ For example, a study of participants in a U.S.-based national educational and screening program for eating disorders reported that comparably symptomatic Latino and Native American participants were significantly less likely than white participants to receive a referral for care, and that symptomatic ethnic minority participants were less likely than whites to have been queried by a doctor about eating and weight related issues.^32^

Diverse social norms also influence whether patients disclose or seek professional intervention for mental health symptoms.^9^,^33^,^34^ For example, culture-specific explanatory models frame expectations for the most appropriate venue to address illness as well as for outcomes.^35^,^36^ Evidence also supports that the perception of clinician sensitivity to cultural background, including concerns about racial discrimination and stereotyping, influences treatment seeking.^37^ Additional studies document lay perceptions of eating disorders that suggest that neither their associated clinical risks nor the most effective treatment options are uniformly appreciated among prospective health consumers.^11^,^38^–^40^ Further evidence suggests that individuals with an eating disorder may seek treatment for weight loss^11^ or for other health problems,^4^ rather than for their eating disorder. Depending on local norms, the social costs of disclosure and help-seeking may be judged to be substantial if a disorder is socially stigmatizing. Mental illness is commonly stigmatized,^41^,^42^ and associated with status loss, discrimination,^43^ and barriers to accessing rehabilitative care.^44^ Several reports suggest that eating disorders are associated with stigma among both health professionals and among the general public,^45^–^49^ and whereas some data suggest that stigma hinders help-seeking for bulimia nervosa and binge eating,^50^,^51^ its impact is incompletely understood.

The objective of this study was to identify and describe how social factors—including social stereotyping, stigma, and social norms—may influence access to care for eating disorders by examining qualitative data from ethnically diverse past and prospective health care consumers.

## Method

### Design

We conducted an exploratory, secondary analysis of a qualitative database comprising cross-sectional interview transcripts collected in 1998 and 1999 (*n* = 32). These qualitative data had originally been collected from a sub-sample of respondents who had previously participated in a quantitative study (*n* = 289) that found ethnic disparities in clinician queries and referrals for eating and weight related concerns.^32^ The follow-up, in-depth interviews had been conducted to supplement these quantitative study data by probing health consumer experience with treatment and its potential relation to ethnic disparities in access to care. This study was approved by the Partners Human Research Committee.

### Participants

Narrative data utilized for this secondary analysis were initially collected from respondents identified by their previous participation in a follow-up study to the 1996 National Eating Disorders Screening Program (NEDSP).^32^,^52^ Eligibility for the interview study had required that respondents had (1) affirmed current or past “concerns, symptoms, or problems” regarding eating or weight; (2) self-reported ethnic identity; (3) provided contact information that was still valid when the sub-sample was defined; and (4) were at least 18 years of age. The sampling strategy included purposive sampling of all eligible ethnic minority respondents to maximize ethnic diversity, which reflected the previous study's aim to examine the relation of ethnicity to access to care. For this previous study, “ethnic minority” participants were defined by self-report of any ethnic or racial identity other than “Caucasian” in a forced choice item on the NEDSP questionnaire. This sub-sample was designated as the “ethnic minority” group (*n* = 12; response rate 67%). The “non-minority” (i.e., Caucasian) comparison group (*n* = 20) comprised a purposive sub-sample identified as “key informants” (*n* = 5; a convenience sample of the first five key informants scheduled) combined with a randomly selected sub-sample from all others meeting eligibility requirements within this “non-minority” cohort (*n* = 15; response rate 44%).

### Procedures

#### Development of the Qualitative Database

Cross-sectional, qualitative data were originally collected by semi-structured, open-ended, telephone interviews (*n* = 32). Item content—designed to probe the relation of ethnicity to barriers to care for an eating disorder—had been adjusted after the five key informant interviews. Interviews—each at least 45 min in duration—were audiotaped and later transcribed. Interview topic content included structured questions about education, employment, ethnic identity, health insurance, and history of treatment seeking for eating and weight concerns. Semi-structured, open-ended questions included items requesting respondents to describe their experiences in both seeking and receiving treatment as well as personal experience with ethnic or racial stereotyping in health care and other encounters. Specific topics addressed decisions to seek care, difficulties encountered, a critical appraisal of clinician empathy and competence, and ethnicity-specific factors that influenced this process.

#### Data Analysis

The study team examined interview transcripts to identify key themes relevant to treatment access and therapeutic engagement. We also extracted self-reported data on demographic and health characteristics relevant to study questions (e.g., ethnic identity, gender, history of an eating disorder, treatment history, and health insurance at the time of interview) and confirmed assignment to “ethnic minority” and “non-minority” sub-groups. Interview transcripts were inputted into NVivo 8^53^ for initial coding, sorting, and quantifying thematic content of interest within the two strata defined as “ethnic minority” and “non-minority.” Two study team members independently identified and coded all occurrences of text relating to stereotyping, social norms that impacted help-seeking, socio-demographic factors impacting communication or empathy in a clinical encounter, and financial constraints and treatment resource availability. Discrepancies in these initial coding assignments were resolved through discussion and/or input by a third investigator and stigma/shame was subsequently coded as another category of interest. Final coding assignments for the key social barriers—social stereotyping, stigma, and constraints associated with availability or affordability of treatment resources—were refined during subsequent evaluation within the context of individual and aggregated interviews. We also identified narrative data and specific excerpts supporting and illustrating respondents' perceived impact of stigma and/or stereotyping on treatment seeking, access, or engagement. Finally, we used the matrix function of NVivo to examine frequencies of these perceived social barriers in the study sample and within the ethnic minority and non-minority sub-samples.

## Results

The study sample was ethnically diverse, including 12 respondents who reported African-American, African-Caribbean, Asian-American (Chinese, Japanese, Korean), Latino, and Native American heritage. Among 20 respondents who self-reported being “white, non-Latino” or “Caucasian,” 10 reported additional details of their ethnic identity. Almost all respondents were female (*n* = 29) and had health insurance at the time of the interview (*n* = 30); approximately, one-half were college age. Most participants also self-reported a current or past eating disorder (*n* = 23), and a majority reported having received some treatment for an eating disorder or “any eating or weight related concerns, symptoms, or problems” (*n* = 24). However, one-quarter (*n* = 6) of the participants who reported a current or past eating disorder had never received specific treatment for it. Study sample characteristics are summarized in **Table****1**.

**TABLE 1:** Selected socio-demographic characteristics of study participants

	Ethnic Minority Participants: *n*(%)	Non-minority Participants: *n*(%)	Total: *n*(%)
Gender			
Female	10 (83.3)	19 (95)	29 (91)
Male	2 (16.7)	1 (5)	3 (9)
Approximate age			
College age	6 (50)	8 (46)	14 (44)
Post college-age	6 (50)	12 (54)	18 (56)
Self-reported ethnicity^a^			
“Non-minority”			20 (62)
White, non-Latino; Anglo, or European ancestry		20 (100)	
“Ethnic minority”			12 (38)
African-American	4 (33.3)		
Latino	3 (25)		
Asian-American	2 (16.7)		
Multiethnic	3 (25)		
Self-report of past or current eating disorder^b^	7 (58.3)	16 (80)	23 (72)
Respondents within this group who never accessed treatment n/N (%)	3/7 (43)	3/16 (19)	6/23 (26)
History of some treatment for an eating disorder or an “eating or weight related concern”^c^	7 (58.3)	17 (85)	24 (75)
Health insurance at the time of the interview	12 (100)	18 (90)	30 (94)

a As chosen from list presented by interviewer and with information supplemented from participant. Each participant is listed in only one ethnic category.

b In response to direct query in interview.

c In response to either direct query in interview or supported by other self-reported descriptions of treatment.

### Perceived Social Barriers to Care

Respondents identified numerous and diverse perceived barriers to accessing care for an eating disorder. Among these, we discerned impediments to care attributed to or associated with social costs, social norms, and ethnic identity, which we classified broadly as “culturally-based barriers.” We also identified societal factors relating to affordability and availability of health services. For the purpose of addressing this study's aims—examining a spectrum of social barriers to care—we distinguished these two categories for analysis, although we recognize their substantial potential to be intertwined. Therefore, for this study, we define “culturally-based factors” as those that fundamentally stem from prevailing values, beliefs, practices, and norms within a specific cultural reference group. In this sense, a broad and inclusive use of the term, “culture,” is intended to encompass mainstream, ethnically-defined, and other locally-defined, as well as hybrid, cultural identities. In contrast, for this study we define “societal factors” as those more related to the distribution of economic and health care resources. Respondents attributed adverse impact of these social factors in the domains of their treatment seeking, communication in the clinical encounter, and/or therapeutic engagement.

### Culturally Based Barriers to Care

#### Stigma and Shame as Perceived Barriers to Care

Descriptive data on stigma and its identified impact on care are summarized in **Table****2**. Over half (*n* = 19) of the respondents reported personal experience with stigma or shame that appeared to have an adverse impact on care. Specifically, respondents perceived that social costs related to acknowledging an eating disorder had influenced them to avoid or postpone treatment or limit their disclosure of related symptoms. For example, concerns about help-seeking or disclosure included that their symptoms would be viewed as a “weakness” or a “character flaw,” would shame or disappoint their families, or would result in a lasting and negative label of mental illness (a “black mark”).

**TABLE 2:** Perceived impact of stigma and/or shame on care for eating and weight symptoms

ID #	Gender	Ethnic Identity	Selected Interview Excerpts and Context Supporting Experience of Shame and/or Stigma^a^	Selected Interview Excerpts and Context Supporting Perceived or Apparent Adverse Impact
Ethnic minority study participants				
10	F	African-American	Spoke generally of what it can be like to be black and seeking care for an eating disorder inasmuch as it generates the feeling that “maybe there's something wrong with your racial identity, maybe you shouldn't be doing this, this is not your problem, type attitudes can be really hurtful. You know to already know that you're doing this, less than healthy thing, not only is your health compromised, but your identity is compromised too. That can be a lot.”	Feels that the embarrassment that black individuals experience having an eating disorder generally may make it more difficult to seek care.
11	F	Asian-American/Chinese	Perceived “less encouragement to seek outside help. [ … ] you were taught to be very subservient and feel shame and guilt [ … ]” This perception specifically applied to disclosing symptoms to non-Asian clinicians, but also applied to seeking any type of counseling, “And you know there was also this stigma, I don't know where it came from, of you know having to get counseling for this stuff was, I don't know, like you're mentally ill—you're a nut. You're a kook.”	Family attitudes kept respondent from seeking care; felt she needed to “sit and live with it.” Avoided talking about eating struggles, and went many years without “asking anybody for anything.”
17	M	Latino; Puerto Rican	Felt that seeking help was “silly” and that eating issues were “something I should deal with in myself” and that “It was more of a personal thing than anybody else helping me with it.”	Resistant to seeking help; eventually sought help on recommendation of others, though “really was hesitant going in the first place”
18	F	African-American	In the black community, talking about weight is a “touchy subject,” so “a lot of times things don't get discussed,” even in the context of obesity-related illnesses.	She stated that she was “[ … ] shy about, or reluctant to talk about” weight concerns with a screening counselor; “Well, usually for me we won't talk to people about my weight.”
21	M	Latino	“[ … ] even hide it from my own family. My mother has said that is a personal, that's a personal thing, and you don't go tell people, especially your friends, about stuff like that. [ … ] Unless it's somebody you can really, really, extremely trust a hundred percent like you can do yourself. Like you do yourself. Like I do myself. But I've never said a word to any of my family or friends about it.”	Admits some limited disclosure with clinicians: “[ … ] I really didn't bring everything up at the time [ … ]” about eating but also expressed his wish that they had asked him more in-depth questions about it. “[They should …] ask the student to be more in-depth about it.” “[If there were more time], I could spill the beans to him more and see what he would say.”
Non-ethnic minority study participants				
2	F	White/Polish, Hungarian and Italian descent	“I mean because it's just, you know, fat people are always looked at as like weak people, sloppy people, you know they don't care about themselves, they don't want to take care of themselves, but that's not true. And it's been a lot of trying to overturn those stereotypes.”	She initially avoided therapy: “At one time I did [avoid care] cause like I said, what my family was saying to me about ‘It’s really not a problem and you're making a mountain out of a mole hill.' ” Concerned that her problem would be seen by health care providers as a lack of “will power.”
			Perceived lack of support from family for care-seeking: “You know my family had a big problem with my going to therapy, […] It was like they felt there was something wrong with them because I had to go to therapy […].”	
3	F	White	Ashamed of lack of control over eating, in context of self-image as a good student, athlete, and “really put-together person.” Reported a “crazy fear” that clinicians “aren't going to take you seriously” and “aren't going to think your problems are as bad as other patients,” or that people would talk about her as “ ‘the girl with that problem.’ ”	She “let it go on for so long” without telling anyone. “But it was like, ‘well I have to deal with this situation and I can't possibly tell anyone about it, so I have to deal with it myself.’ ” She felt symptoms became entrenched and “a coping mechanism for everyday life.” She eventually sought help.
4	F	White/Jewish	Embarrassed about overeating. “I'm just embarrassed.”	Does not discuss symptoms. Embarrassment kept her from talking to a friend whom she believed would be supportive and possibly knowledgeable about the problem. She also was reluctant to seek support via OA: “[ … ] the counselors at the hospital were trying to get me to go to OA and I wouldn't go. And I wouldn't go because of the name and what it meant [ … ] I'm not going to go to something that has a horrible name [ … ].”
7	M	White	Embarrassed by what doctor might say: “[ … ] you want to pass the test; you want to look great, and kind of hide the truth.”	Did not discuss weight concerns with physician; preferred to talk to counselor or family.
12	F	White/German and Irish descent	Reluctant to disclose information: “Not, not letting go of too much information, not making my weakness very vivid, because I didn't want to be taken advantage of or to be laughed at or anything…. I was very hesitant to talk about it at first.” And “[ … ] so then telling somebody about it was more just like saying, ‘well yeah, I'm weak. And admitting that, and it just felt bad.’”	Generally reluctant to disclose information about eating disorder symptoms. Postponed treatment for several years after she was aware of the onset.
14	F	White/Polish descent	Family considered seeking treatment a weakness: “[ … ] my parents said, ‘well if you tell people, then they are not going to let you play tennis and they are not going to let you do this,’ so I had a bit of fear in me that they weren't going to let me participate in sports anymore, that I was going to have this stigma surrounding me.”	Dissuaded from seeking treatment because: “[…] it was just the fear that my family kind of put into me that it's a bad thing—it's going to hurt you for the rest of your life if it ever goes on your record that you've had this problem.” Parents asked her not to seek specialty care for the eating disorder: “Because I would have a record. And if I ever wanted a job in the future, they would see that and basically the whole stigma thing.”
15	F	White	Admits limiting disclosure with peers: “I think that it's not to really talk about it. I mean, I do to a certain point, but there's OK places to talk about it and not OK places to talk about it.” And with her clinician: “I'm not always like truthful about things. I just usually, I don't say anything about it. Like if something's bothering me or I don't want to say it cause it's like, I don't know, something's that's probably needs to be known but I'm afraid that, it's like embarrassing for me to talk about, I won't talk about it.”	Perception of social acceptability limits disclosure to peers; embarrassment limited disclosure to a clinician.
20	F	White	Embarrassment over eating issues as a “character flaw” in otherwise high-achieving self-perception. “And so you're embarrassed to ask what other people think or if you should go get help.”	Uncomfortable discussing eating issues. Delayed seeking care: “I wouldn't go at first;” but later sought care after deciding “[an eating problem is] embarrassing and it's ugly, but it's not a big deal.”
22	F	White	Reluctant to disclose weight concerns to friends: “I, I just, I was ashamed about it. I didn't think that they would understand.” as well as to professionals: “I felt awkward about [asking for professional help for weight concerns].”	Reluctant to seek support and professional help in high school; felt able to seek professional help in college.
24	F	White	Eating/weight concerns are “personal business” and an uncomfortable topic to discuss. “I really don't like to talk about it at all.” “I don't sit down, I wouldn't pull someone aside and just say, ‘I need to chat about my weight.’ That's just something I wouldn't do.”	Limited help-seeking: Brought up eating/weight concerns once with primary care doctor, but is unlikely to do so again because of “not liking to talk about it.” Sought therapy and medication for nonweight issues, but did not discuss eating or weight with those clinicians.
25	F	White/Hungarian descent	Embarrassed to discuss dieting. Sense that others aren't concerned about her eating habits; they don't understand: “Cause people aren't, they're not that concerned with it right then and there. And they don't understand, either.” Also: “Some people, if they, they just think you don't have enough will power. Or they just think you're lazy. Do you know what I mean? Like some people won't take it as like a medical problem.”	Initial non-disclosure (except sometimes to mother) about eating habits to clinicians when she was younger: “I don't think I would really know to go to the doctor and ask the doctor. I was probably embarrassed to go to the doctor and talk about it.”
27	F	White/Irish-Catholic	Experienced shame and discrimination related to her weight: “And it was looked upon if you were obese you were glutton. You're a sloth.”	Nonspecific impact on relation with her clinicians; for example, she stated: “You know I made sure I looked OK even though I was huge. It was like dressing up on the outside to hide the [expletive deleted] on the inside ’n stuff. And I carried it off pretty good.” Also describes difficulty in self-disclosure due to shame: “I get sick to my stomach knowing I have to go in and tell him what's been going on. Yeah, it's not easy.” and admits to limiting disclosure initially in treatment.
28	F	White/Italian/French-Canadian descent	“I was, it was a perfect combination of ashamed and feeling like no one would do anything or could do anything about it. It wasn't an issue that other people could help you with, I thought you know, you just got to buck up and eat better.” Family did not support her seeking treatment for this problem. “I mean I don't think that my family's really big on going to the doctor's unless you were dying, you know? And you know weight concern is more like a moral issue. In a way, make it like, ’You eat too much,' or ’Don't be such a pig and you'll be fine with your weight' kind of thing.”	Many years delay in seeking treatment.
30	F	White/French and German descent	“Afraid they [clinicians] would think I was certifiable and put me in a funny farm.” She stated: “If you want a big black mark against you, go to the psychiatric ward.”	Fear of hospitalization led to non-disclosure to clinicians about emotional reasons for restricting food; she stated: “It's a pretty good motivator to keep your mouth shut.”
			Also: “I still think it is sort of a taboo subject. It is just not something that people talk about. Umm…. It is just not recognized as a problem. It is some sort of personal weakness if you can't deal with these things. If you are fat it is a personal weakness. A character flaw.”	

a Direct quotations from study participants are placed in double quotation marks; bracketed ellipses denote nonessential text removed for streamlined presentation; investigators' interpretation of pronouns or implicit context placed in square brackets.

#### Social Stereotypes as Perceived Barriers to Care

Six respondents perceived that social stereotypes concerning eating disorders or weight had a personal adverse impact on help-seeking, care, or other support (**Table****3**). Specifically, respondents perceived that concerns or symptoms had been unrecognized, misinterpreted, or dismissed because of expectations about the presentation of an eating disorder or social norms relating to weight. Although both ethnic minority (*n* = 2) and non-minority (*n* = 4) respondents perceived stereotypes relating to ethnicity and eating disorders as impeding access to appropriate care, their experiences of this impact were qualitatively distinct. For example, non-minority respondents reported the perception that symptoms were dismissed or trivialized but mostly perceived that this was because family members or clinicians viewed eating or weight concerns and symptoms as socially normative rather than as clinically concerning. One non-minority respondent reported that the assumption that she had an eating disorder—because she was young and female, and possibly also because she was white—delayed an appropriate referral for a gastrointestinal illness. Conversely, another non-minority respondent indicated that a social stereotype that eating disorders are “rampant” among white girls had actually facilitated help-seeking. She observed that, “[…] in that culture it's more acceptable to have eating disorders so you, maybe I have felt that I didn't have to hide it;” concluding that, “I guess it has made it easier to talk about umm… easier to admit.” [ID 26]

**TABLE 3:** Selected interview excerpts illustrating social stereotypes relevant to weight and eating concerns and their perceived impact on care

ID #	Gender	Ethnic Identity	Source	Excerpt and/or Context Reflecting Social Stereotype^a^	Perceived or Apparent Impact on Care
Ethnic minority subjects:					
10	F	African-American	Clinician Social network	Black girls perceived not to engage in disordered eating: “For years [vomiting] went on, but no one ever looked at me because, you know, white girls do that, not black girls. It was one of the easiest things I ever gotten away with in my life.”	Eating disorder was not recognized for several years and she felt it was not properly attended to once recognized; reported poor engagement in treatment and eventually ceased treatment because of this.
					Describes difficulties she has encountered in having her illness taken seriously: “I think it makes it more difficult because I have more trouble having people listen to me. I've had a lot of black women tell me that black women don't really have eating disorders, eating problems, however it's phrased. So it's kind of a struggle. [ … ] But I don't think people are willing to listen because in a lot of people's minds its a white person's problem and it's not.”
23	F	Multi-ethnic (including African-American)	Clinician	Psychologist did not believe she had an eating disorder because she was black: “[ … ] her comment to me was she did not believe that I had an eating disorder because I don't fit the stereotype.”	“Well, when I saw her, I explained to her things that had happened, but at the same time, I felt like basically what I was saying was going over her head, because it was like it was almost going in one ear and out the other, she wasn't paying any attention to me or taking her job seriously because she just didn't think that I was telling her the truth.” The psychologist: “said that she didn't know how she would be able to help any further because she didn't feel that I fit the stereotype or the protocol with someone with an eating disorder.” Subsequently, she left treatment with that therapist and felt more wary about discussing her eating disorder.
Non-minority subjects:					
2	F	White/Polish, Hungarianand Italian heritage	Social network	Symptoms were not perceived as problematic: “As far as having you know, having like I said the Polish and Italian background. I figured it was more of what was expected. Instead of being thin [ … ] people want you to eat, they want you to be healthy, because eating is healthy, you know, ‘oh, you're a big girl, you're healthy.’ [ … ] I mean, I just, I kind of felt alone. And I felt like I shouldn't be saying anything. You know, I felt like I should just accept it, and you know, just go on, because no one else thought it should be a problem, so why should I think it's a problem. And I was making a whole bunch of other problems by bringing it out. You know? And um it was just really difficult getting people to understand what I really wanted, you know that I didn't want to be this weight, that I didn't want to be a big girl.”	She felt that she had to overcome lack of recognition of overeating and overweight as symptoms, and that she had to be extraordinarily proactive about seeking treatment. “I was pretty much the one that got my own help. I mean, you know it wasn't like someone looking at me like saying, ‘I think you have a problem’ and for me it was harder because I wasn't like stick-thin.” Once she sought treatment, opposition and lack of understanding complicated the implementation of changes.
8	F	White	Clinician Social network	She encountered a lay stereotype “ ‘Oh, you know you're a young female; you're just automatically concerned about your weight.’ ” and a clinician stereotype: “They just assume that you know you're a young girl and whatever you have an eating disorder.” She acknowledged (but was uncertain) a possibility that ethnicity may have influenced her clinician's assumptions, “unless he just saw me as just another, you know, white girl going through, you know, eating problems, whatever, weight problems and all.”	Delayed receiving appropriate treatment for GI symptoms because a clinician assumed she had an eating disorder and did not evaluate other possibilities. She had hoped clinicians would respond in a way such as: “we're gonna try to you know get to the bottom line here,” but it wasn't like that. It was like, ‘Ugh, you know, another one,’ you know type-deal, and I was like this isn't, this isn't what I'm looking for, so.”
28	F	White/Italian, French-Canadian/Catholic heritage	Clinician Social network	You have to be emaciated or obese to have an eating disorder. “[ … ] unless you know you were on one end of the spectrum or the other, you couldn't address it.”	Symptoms not recognized or taken seriously, especially by Italian family, which tolerated greater weight fluctuations. Difficulty disclosing symptoms: “[ … ] it's really hard to be taken seriously unless you know become completely anorexic or completely obese in terms of like eating stuff … people look at you and like, ‘What's your problem?’ you know, ‘you're fine.’ ”
30	F	White/French and German heritage	Social network	Stated that a difficulty she encountered in trying to get help for eating and weight concerns related to her feeling: “That people don't see it as a concern. That it's a character flaw. That it's a weakness.” Stated that “You know, the general perception [of friends and clinicians] has been that eating disorders are—women who suffer from eating disorders are vain, selfish, petty little creatures who are focused only on their weight and physical appearance, when it's just a symptom of deeper problems.”	Did not seek treatment for eating disorder, which began before college. Eating concerns were not addressed until she received treatment for another mental illness. She never told her family of origin that she had an eating disorder.

a Direct quotations from study participants are marked; other text is investigator interpretation based upon interview content.

By contrast, two ethnic minority respondents, both identifying African-American heritage, perceived that their symptoms had been dismissed because a clinician believed that eating disorders did not affect African-Americans. These respondent narratives identified an explicit, central, and adverse impact of racially-based stereotypes on their care. Specifically, they described experiences with clinicians who had dismissed or disregarded symptoms of an eating disorder, apparently because they did not expect them in an African-American patient. For example, one of these participants alluded to disconfirming feedback about her symptoms from her social network in her statement “[ … ] I am not supposed to have the eating condition that I do” and its influence on her disclosure of information about her eating disorder. She also revealed that her psychologist had not believed that she had an eating disorder because she did not fit the “stereotype.” She recounted, “[ … ] once she said that, I basically kind of said, OK and walked away…” In addition to her description of leaving treatment with this therapist, she explained:

“I'm a little bit worried about who I talk to, especially about the eating disorder now, but for other things too. Based on the incidents with the psychologist, that's the reason for that.” [ID 23]

The other respondent's narrative reflects her view that her clinician appeared to be poorly informed and to have little interest in understanding the relation of her ethnic identity to her illness. She explained,

“[ … ] I was very much aware that she [the clinician] knew very little about my culture and she wasn't asking me, ‘Well, what does food mean to me in my family, what is it like for you, let me understand better your world,’ she never did that.” [ID 10]

This respondent described her irritation by this insensitivity, as well as its impact on her decision to leave treatment, as illustrated in the following excerpt from her interview:

“Yeah, because I'm not just a darker version of the prototype, I've got some things, you know that might be different than what she's experiencing going on in my life. So ask me. Find out what my life is like. What this means for me, what's so difficult for me. [ … ] but she never did that. I was pretty disgusted with her. I stayed a year, I tried.” [ID 10]

She reflected further on her clinical encounter with stereotyping in her statement,

“Yeah, I think [my clinician] put me into the generic mold, which happens to be the white mold, and I don't even know if that same white mold applies to some of the white people I encounter.” [ID 10]

#### Social Norms and Socio-Demographic Distance as Perceived Barriers to Care

Ethnicity-specific norms for both help-seeking and healthy weight appear to be factors that dissuaded respondents to seek care. For example, a female respondent of Chinese heritage spoke of the message she received against seeking help, especially from a non-Chinese clinician:

“The desire to ask outside the race was kind of like, ‘Oh, well you don't want them to know that about you.’” [ID 11]

And a white, non-Latino female respondent whose family had lived in the United States for many generations said that weight “[ … ] is just not what polite people talk about.” [ID 30]

In addition, perceived social costs to breaching ethnic identity or evincing a stigmatizing illness potentially moderate effects of social norms for treatment seeking. An African-American respondent explained:

“You know, to already know that you're doing this, less than healthy thing, not only is your health compromised, but your identity is compromised too. That can be a lot.” [ID 10]

One white, non-Latino respondent feared,

“[I]t's going to hurt you for the rest of your life if it ever goes on your record that you've had this problem.” [ID 14]

Respondents also perceived that socio-demographic factors (including age, gender, and social background) had resulted in suboptimal communication, clinician attunement, or therapeutic engagement in clinical encounters. In fact, a majority of participants (53%; 13/20 non-minority, 4/12 minority) perceived actual or hypothetical barriers to therapeutic engagement related to these socio-demographic differences, although some of these were not necessarily specific to care for an eating disorder. These perceptions are illustrated in the following interview excerpts (all from non-minority respondents):

“And then they kind of talk to you in like that condescending kind of tone, like they are just tolerating you, like they're, you're lucky to have their time to sit there and for them to listen to you. You know. And then with women, it's so different.” [ID 2]

“You don't want to talk about your faults with someone from the other sex.” [ID 20]

“I guess I sort of felt that, ah, she kind of dismissed some of my, my ah, feeling or whatever was going on as just things that have to do with my age, things like that.” [ID 26]

“I've always felt very different from the clinicians I've been dealing with and you know, not taken seriously whether it's because of gender or whatever.” [ID 28]

“[ … ] I have to be honest, I didn't like reveal any information. It was sort of like me trying to give the most brief answers as I could. You know, and not share anything more than I wanted to with him. Because I didn't want to be like you know, ‘can I have a woman?’ You know, I didn't want to insult him, but I was like, ‘I'm not sharing anything like this with you.’” [ID 3]

“I mean you can't describe some of these things to a guy, because he just has no frame of reference.” [ID 30]

“There was no…connection there. And he was a male. I had a real problem confiding because he was a male.” [ID 5]

### Societal Barriers: Availability and Affordability of Health Care

Finally, additional impediments to accessing care included economic or health insurance constraints as well as suboptimal availability of specialty services. Respondent excerpts reflect that these financial and geographic constraints had also impacted their access to care. Select excerpts illustrate respondents' experience as follows:

“I think access to care can be a big problem. Even if it's not just a factor of, ‘I don't have insurance, I don't have money,’ finding places at least here where I live that treat eating problems is hard. There are not a lot of places here.” [ID 10]

“Being able to listen to the patient and just giving the patient enough time, you know, that is, of course you're not going to spend two hours in there, [ … ] sometimes I'm out of there in like fifteen minutes. I mean, what can you do in fifteen minutes? You know, it's really ridiculous! You wait all that time in the waiting room, and you know you walk in and he's just rushing you out the door [ … ]” [ID 2]

“And then also my insurance wouldn't cover it any more, so I kinda got kicked out, too.” [ID 4]

“I mean with our medical insurance though, I had to keep my doctor at home. It was really a hassle, because it was an HMO. It was a hassle to switch around the doctors, and things. So, I still had to go home to go to the doctor. And it was two and a half hours away. So that was difficult. You know, if the doctor had been closer I think it would have been a little easier.” [ID 6]

“Even though the counselor was incredibly cheap, I was incredibly broke, so I didn't want to go and pay for them, the counseling, and I just had decided to use my friends in that respect.” [ID 7]

“I really didn't have the means to go like seek a real counselor or [ … ] I guess at one point in time I thought about seeing like the Jenny Craig counselor or some type of counselor, but I couldn't afford that.” [ID 13]

“Insurance is another thing. They always don't like pay for everything that you want them to.” [ID 15]

“Like I couldn't attend that group cause my health plan wouldn't pay for that.” [ID 26]

**FIGURE 1:**
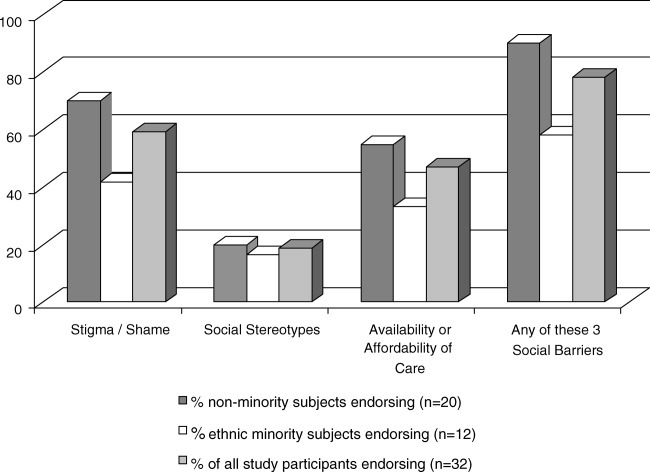
Percentages of study participants endorsing selected social barriers to eating or weight related care in the overall sample and ethnic minority and non-minority sub-samples.

Following Andersen and Newman's (1973) typology of determinants of health care utilization, the availability and affordability of health care appear to function as “enabling components” that mediated access to health services in our study sample.^9^ Fifty-five percent of non-minority participants and 33% of ethnic minority participants endorsed at least one of these societal barriers (**Figure** **1**).

## Discussion

Many individuals with an eating disorder do not access appropriate specialty care, yet barriers to treatment access remain inadequately understood. Although previous research has primarily emphasized psychological factors undermining motivation for, and engagement in, care, this qualitative study identified and examined several social factors perceived to undermine receipt of care for eating disorders and related symptoms. In this ethnically diverse study sample of health care consumers, stigma/shame and social stereotyping were identified as impediments to care for eating disorder related symptoms in both ethnic minority and nonminority groups. These social barriers overlap conceptually with one another and are not necessarily mutually exclusive with additional psychologically-based barriers to care. Moreover, nearly half of study participants also identified cost and availability of health services—frequently invoked factors contributing to patterns of health care utilization^54^—as imposing barriers to specialty care for eating and weight related concerns.

This study augments and complements previous findings by presenting health care consumer perspectives addressing the personal impact of social stereotypes, stigma, and shame on care for eating disorders and related concerns. In comparison with quantitative approaches, these qualitative data also offer a more granular perspective on ways in which social factors discouraged help-seeking or undermined therapeutic engagement for an eating disorder. In addition, these narrative data suggest that stigma, shame, local social and cultural norms, and stereotypes about eating disorders may interact in complex ways that compound their adverse impact on care.

Two respondent narratives that articulated demoralization and frustration related to clinician-held, racially-based stereotypes that appear to have undermined care warrant special attention. Clinicians are trained to consider social and demographic context in evaluating patients, so it is plausible that clinical stereotyping may partially result from an intention to apply probabilistic data in order to enhance efficiency and identification of high risk individuals.^55^ In fact, these findings complement two experimentally based studies that demonstrated how ethnicity influenced clinician and lay expectations about the presence of an eating disorder in a fictional patient.^56^,^57^ Although application of socio-demographic risk data may augment clinical data in ways that are potentially helpful to making a prompt diagnosis in some cases, its pre-emption of a complete diagnostic evaluation is problematic. Moreover, data regarding demographic risk correlates of eating disorders are themselves subject to many limitations. Ethnic diversity in help-seeking patterns and in presentation may contribute to bias in prevalence estimates in relation to ethnicity across illnesses.^19^,^58^ Clinical prototypes derived from homogeneous and unrepresentative study populations may be self-perpetuating.^59^,^60^ Although clinicians need to understand the full social context in which an eating disorder presents, inference based solely on ethnicity is unacceptable practice.^61^ Taken together, these findings suggest that racial stereotypes may affect the recognition of eating disorder symptoms and highlight the importance of educating clinicians about potential clinical biases that might hinder detection of eating disorders in ethnic minority patients.

Study findings that stigma and/or shame undermined treatment-seeking for eating disorders are also noteworthy and resonate with research reports that have identified stigma attached to eating disorders by both clinicians and laypersons.^45^–^49^ Although stigma broadly relates to care-seeking across other mental disorders,^62^ clinicians should be mindful of vulnerability among prospective health care consumers with an eating disorder as well. Shame affecting treatment seeking for an eating disorder may also reflect individual psychological attributes^63^ that moderate the impact of prevailing social norms. Although we found that stigma and shame impact both minority and non-minority respondents, narrative study data also suggest ways in which ethnicity may mediate the impact of stigma on care.

Other investigators have suggested the value of enhancing clinician understanding of how low “mental health literacy” may undermine treatment-seeking for eating disorders.^38^ This study's findings suggest additional opportunities to consider health consumer perspectives to inform efforts to remove barriers to care. For example, our data support that social stereotypes—when they do occur—may diminish opportunities to either seek or receive care and also undermine therapeutic engagement among both ethnic minority and non-minority patients. Although the majority of respondents in this study did not endorse the view that stereotypes had an impact on care, the occurrence of instances within this small study sample warrants further investigation of social barriers to care related to race and ethnicity in larger, more representative study samples. Future inquiry should also examine professional education strategies to support clinician sensitivity to occurrence of eating disorders in diverse populations. The development of training strategies to improve clinician awareness and attunement to eating disorders would potentially benefit patients from all cultural backgrounds

## Limitations

Study findings should be interpreted relative to several important limitations. The sample is small in size and not representative of the U.S. general population. Albeit small, the magnitude of this sample size is typical of qualitative studies, and was also likely adequate to address study objectives.^64^ Our sample was also limited by the extant qualitative data base and contained very few male study participants. Moreover, study participant recruitment for development of this data base had yielded a low response rate. In addition, a majority of respondents had health insurance and all had attended an educational and screening program for eating disorders held on U.S. college campuses. As a result, the prevalence and distribution of social barriers identified in this study sample is unlikely to be representative of—and cannot necessarily be generalized to—other populations. Moreover, social norms are fluid and dynamic; thus, stereotypes and stigma perceived at the time the interviews were conducted cannot be presumed to be unchanged. These retrospective data are also subject to recall bias. Therefore, we emphasize that data support perceptions of barriers to care rather than objective impact on care or outcomes. However, we also suggest that data from interviews probing retrospective experience may be especially informative when enriched by greater maturity or insight with age and experience. We also assert that the inclusion of subjective data represents a distinctive strength of this study, because patient voices are underrepresented in the research literature on eating disorders, yet merit attention in order to complement and inform quantitative data based findings.

Next, the eligibility criteria for the study permitted respondents with either eating or weight-related concerns. Consequently, some of the perceived barriers identified in this study may not be specific to eating disorders care, but rather may relate to treatment access for a broader spectrum of eating and weight-related concerns with diverse clinical significance. The resulting diminished specificity is a limitation arguably offset, however, by its inclusion of participants who may not have recognized how their symptoms relate to an eating disorder. In other words, prospective mental health consumers often must decide whether and where to seek treatment without the benefit of a fully informed understanding of the clinical relevance or the most effective therapeutic options for their problem.^11^,^38^–^40^ Moreover, consumers may prefer initially to seek care for related weight or other medical concerns.^5^,^6^,^11^ As a result, the clinical diversity of the study sample potentially provides a broad perspective on barriers encountered by patients with an eating disorder.

Finally, our operational definition of ethnic minority was limited. Ethnic identity is a complex construct and our interview probes may not have fully tapped its multidimensional impact on care. In addition, we had an insufficient number of study participants to allow examination of ethnic heterogeneity within either minority or non-minority groups in this study. Ethnic identity and descriptors in the United States cannot be extrapolated to other social contexts. Notwithstanding acknowledged limitations of qualitative data for drawing causal inference, they enlarge the scope of understanding of complex social phenomena and complement statistical data in elucidating the context of behaviors within personal narratives.

## Conclusion

This study identifies and characterizes potential social barriers to care for eating disorders and related concerns from a health consumer perspective. Social factors identified in this study sample included stigma and social stereotypes that were perceived to interact with social norms for help-seeking as well as communication and response to symptoms in clinical encounters. These social barriers have impact on both ethnic minority and nonminority health consumers. Societal barriers—that is, constraints related to affordability and/or availability of health care services—were also frequently perceived barriers to care. These study findings augment previous reports relating stigmatization and ethnicity to underutilization of care for eating disorders. It is noteworthy that socio-demographic factors in addition to ethnicity, including age and gender, were perceived to impact care-seeking and experience in the therapeutic encounter. Although similar social factors may have broad impact on inequities of health care access and distribution, study data also suggest potential specific vulnerabilities among consumers with an eating disorder. Social impediments to care may be suitable targets for future interventions to promote optimal and equitable care access for eating disorders. Future research should examine how psychological and social barriers may interact to undermine treatment seeking as well as the prospective impact of enhanced clinician sensitivity to these barriers to care.
